# Supplementary material for: Directional translocation resistance of Zika xrRNA
Source: Nat Commun. 2020 Jul 27;11:3749. doi: 10.1038/s41467-020-17508-7 (PMC7385498; doi:10.1038/s41467-020-17508-7)
Supplement: Supplementary file 1 — Supplementary Information [file 41467_2020_17508_MOESM1_ESM.pdf]

Supplementary information
Directional translocation resistance of Zika xrRNA

A. Suma *et al.*

(Dated: June 13, 2020)

Supplementary Methods

A. Mapping simulation units to physical units

SMOG interaction parameters are in reduced, or simulation units that need to be appropriately mapped or rescaled to match the behaviour of the simulated molecule at the physical temperature of interest.

For our system, which is studied at a single temperature, the mapping of various physical quantities is conveniently expressed in terms of a single rescaling parameter, α, as summarised in the table below.

	quantity in simulation units	quantity in physical units
length	$\sigma^{\rm SMOG}$ [Å]	$\sigma = \sigma^{\rm SMOG}$ [Å] (no rescaling)
mass	$m^{\rm SMOG}$ [g mol^{-1}]	$m = m^{\rm SMOG}$ [g mol^{-1}] (no rescaling)
Boltzmann constant	$k_B^{\rm SMOG}$ [Kcal mol^{-1} K^{-1}]	$k_B = k_B^{\rm SMOG}$ [Kcal mol^{-1} K^{-1}] (no rescaling)
temperature	$T^{\rm SMOG}$ [K]	$T = \alpha\, T^{\rm SMOG}$ [K]
thermal energy	$k_B^{\rm SMOG}\, T^{\rm SMOG}$ [Kcal mol^{-1}]	$k_B T = \alpha\, k_B^{\rm SMOG} T^{\rm SMOG}$ [Kcal mol^{-1}]
interaction energies	$\epsilon^{\rm SMOG}$ [Kcal mol^{-1}]	$\epsilon = \alpha\, \epsilon^{\rm SMOG}$ [Kcal mol^{-1}]
force	$F^{\rm SMOG}$ [Kcal mol^{-1} Å^{-1}]	$F = \alpha\, F^{\rm SMOG}$ [Kcal mol^{-1} Å^{-1}]
MD timescale	$\tau_{MD}^{\rm SMOG} = \sigma^{\rm SMOG}\sqrt{m^{\rm SMOG}/\epsilon^{\rm SMOG}}$ [fs]	$\tau_{MD} = \alpha^{-1/2}\, \tau_{MD}^{\rm SMOG}$ [fs]
force ramping rate	$r^{\rm SMOG} = \Delta{\rm force}^{\rm SMOG}/\Delta{\rm time}^{\rm SMOG}$ [Kcal mol^{-1} Å^{-1} fs^{-1})]	$r = \alpha^{3/2} r^{\rm SMOG}$ [Kcal mol^{-1} Å^{-1} fs^{-1})]

Supplementary Table I: Summary of how different physical quantities are mapped from simulation units (LAMMPS implementation of the SMOG model) to actual, physical units. k_B is the Boltzmann constant. Forces in Kcal mol^{-1} Å^{-1} units can be converted to pN by multiplying by 69.5pN mol ÅKcal^{-1}.

B. Temperature-based calibration

For our system, the rescaling parameter α can be set by calibrating the mechanical unfolding of helix P4, which is 14-nucleotides long, against the known stretching response of RNA helices.

In stretching experiments RNA helices unfold at critical forces of about $F_c = 15$pN at ambient temperature, $T = 300$K, equivalent to thermal energies equal to $E_t = K_B\, T = 4.1$pN nm.

The ratio $E_t/F_c = 0.273$nm defines a lengthscale that can be used to calibrate α, since lengthscales are conserved from simulation to physical units.

To this end, we considered various values of $T^{\rm SMOG}$ and, for each of them, simulating the mechanical unfolding of the helix P4 by pulling apart its terminal P's (atoms indices 1225 and 1505 of PDB entry 5TPY) with a force $F^{SMOG} = K_B\, T^{SMOG}/0.273{\rm nm} = (T^{\rm SMOG}/T)F_c = \alpha\, F_c$. We then looked for the temperature at which folded and unfolded states were equally populated at this applied force, as expected at criticality.

As shown in Fig. 1, the matching condition occurred for $\alpha = T/T^{\rm SMOG} = 3.16$. This scaling value is in the same range as those reported in ref. [1], where a similar calibration procedure was used.

Note that, as summarised in Table SI, this mapping also dictates that, like temperature, the reduced energies and forces of the SMOG model must also be multiplied by the same factor α to have them in physical units.

Force-ramped translocation and stretching simulation were carried out at various equispaced reduced model temperatures which mapped to the following rounded physical temperature values: $T = \{281, 287, 294, 300, 306, 313, 319\}$K.

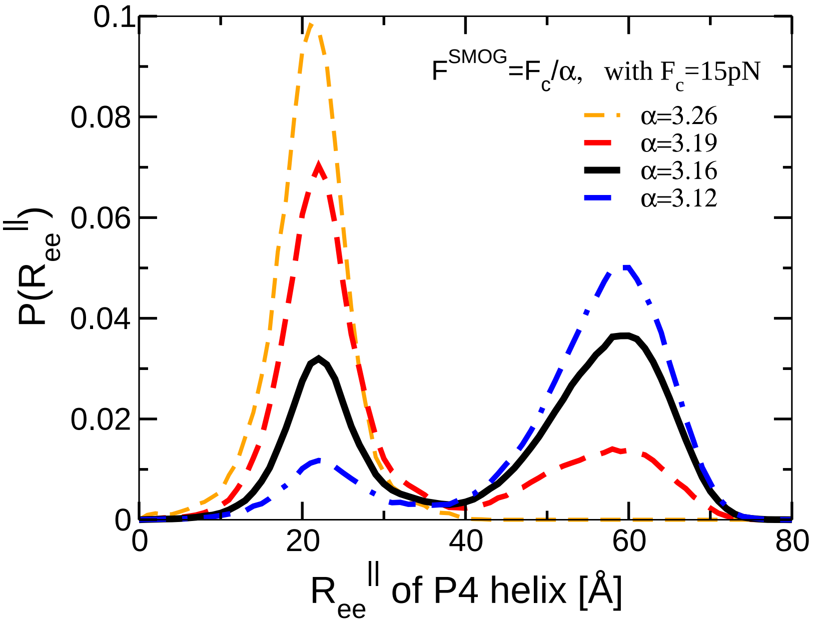

Supplementary Figure 1: Probability distributions of the longitudinal end-to-end distance of the P4 helix in stretching simulations carried out at the indicated values of $\alpha = T/T^{\rm SMOG}$. The stretching simulations were carried out by pulling the terminal P's of the P4 helix (atoms 1225 and 1505 of PDB entry 5tpy) in opposite directions with a force equal to $F^{\rm SMOG} = \alpha{\cdot}F_c$, where $F_c = 15$pN, see text. The data were collected over several trajectories, each covering in total from $2.4{\times}10^8$ to $5.9{\times}10^9$ timesteps at each temperature and force. A balance of folded and unfolded simulations, indicative of the correct combination of temperature and critical force, is observed at $\alpha = 3.16$.

C. Mapping of time

A characteristic time for molecular dynamics simulations is given by $\tau_{MD} = \sigma\sqrt{m/\epsilon}$, where σ and ϵ are the typical values for the range and strength of the interaction potentials and m is the typical mass of the particles. For our model, where we used the actual atomic masses, $m \sim 15$amu, $\sigma^{\rm SMOG} = 4$Å and $\epsilon^{\rm SMOG} = 0.1$Kcal mol^{-1}, which yields $\tau_{MD}^{\rm SMOG} \sim 2.3$ps.

The Langevin dynamical evolution was integrated with the default integration time step of $\Delta t^{\rm SMOG} = 2$fs, corresponding to $\Delta t^{\rm SMOG} = 8.7 \times 10^{-4} \tau_{MD}^{\rm SMOG}$ and with inverse damp coefficient equal to $(\gamma^{SMOG})^{-1} = 2{\rm ps} = 0.87\tau_{MD}^{SMOG}$.

The MD times in reduced SMOG unit can be transformed to physical units by multiplication by $\alpha^{-1/2}$, see Table SI. Accordingly, $\Delta t = \alpha^{-1/2}\,\Delta t^{\rm SMOG} \approx 1.1$fs and $\gamma^{-1} = \alpha^{-1/2}\,(\gamma^{SMOG})^{-1} \approx 1.1$ps. Instead, the force ramping rates in reduced units are transformed to physical units by multiplication by $\alpha^{3/2}$, see Table SI.

We recall that mapping of time from reduced to physical units is only approximate since the absence of explicit water molecules in the model might artificially accelerates the dynamics, see e.g. ref. [2].

D. Parameter combinations for translocation and stretching simulations

	r/r_0	T [K]
1	0.3	300
2	1	281
3	1	300
4	1	319
5	5	300
6	10	281
7	10	294
8	10	300
9	10	306
10	10	319
11	20	300
12	50	300
13	100	281
14	100	287
15	100	294
16	100	300
17	100	306
18	100	313
19	100	319

Supplementary Table II: List of the 19 combinations of force-ramping rates and temperatures used to profile the translocation response. For each combination we carried out 20 simulations from either of the two ends.

	r/r_0	T [K]
1	1	281
2	1	300
3	1	319
4	10	300
5	100	281
6	100	287
7	100	294
8	100	300
9	100	306
10	100	313
11	100	319

Supplementary Table III: List of the 11 combinations of force-ramping rates and temperatures used to profile the mechanical stretching response. For each combination we carried out 20 simulations.

Supplementary Discussion

E. Translocation and stretching behaviour

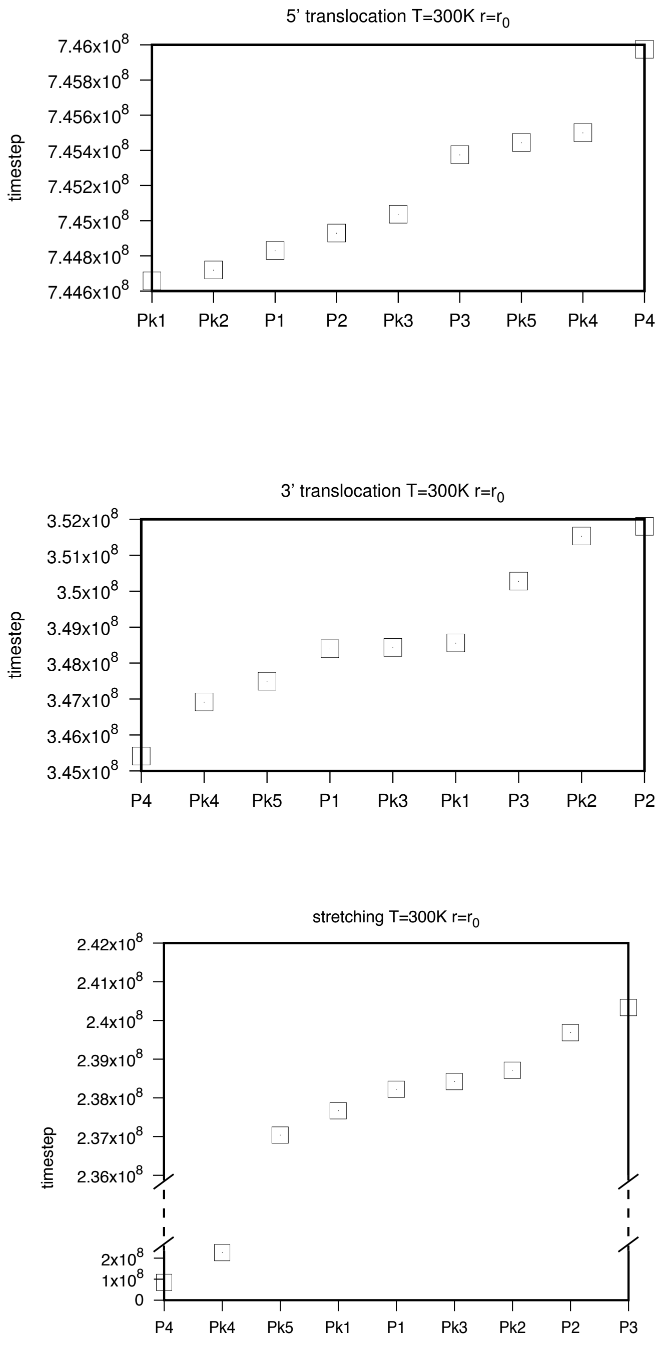

Supplementary Figure 2: Rupture times of secondary elements during three trajectories: translocations from the $5'$ and $3'$ ends, and stretching. The rupture event of each secondary element was defined by the fraction of native contacts (overlap) falling below 0.02.

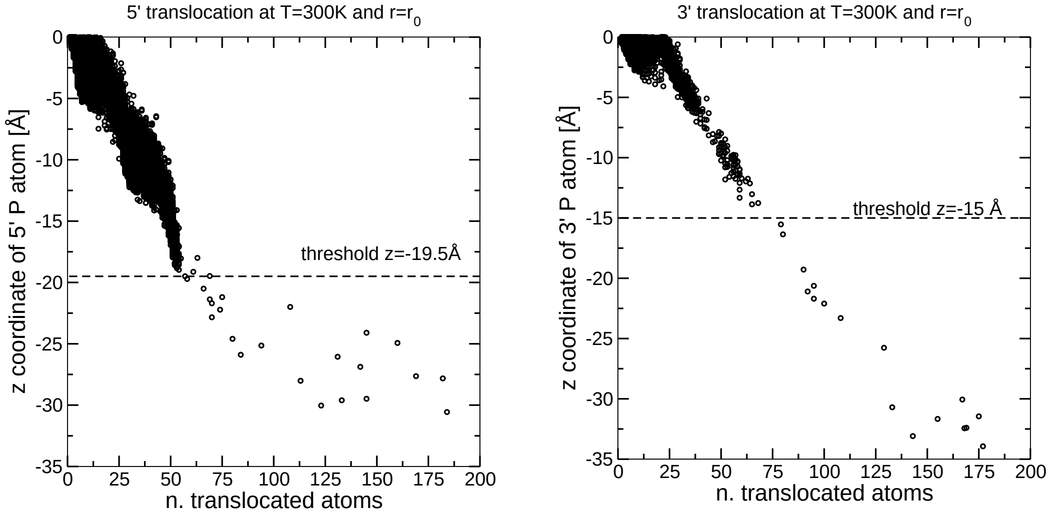

Supplementary Figure 3: Scatter plot of the z coordinate of the P atoms at the $5'$ and $3'$ end versus the number of translocated atoms for force-ramped translocations at $T = 300$K and ramping rate, r_0. In both cases data are for a single run and are, for clarity, limited to the initial stages of translocation. The onset of irreversible translocation is clearly visible as a sparser distribution of the points in the graph. The z thresholds defining the triggering of irreversible translocation at the two ends are highlighted.

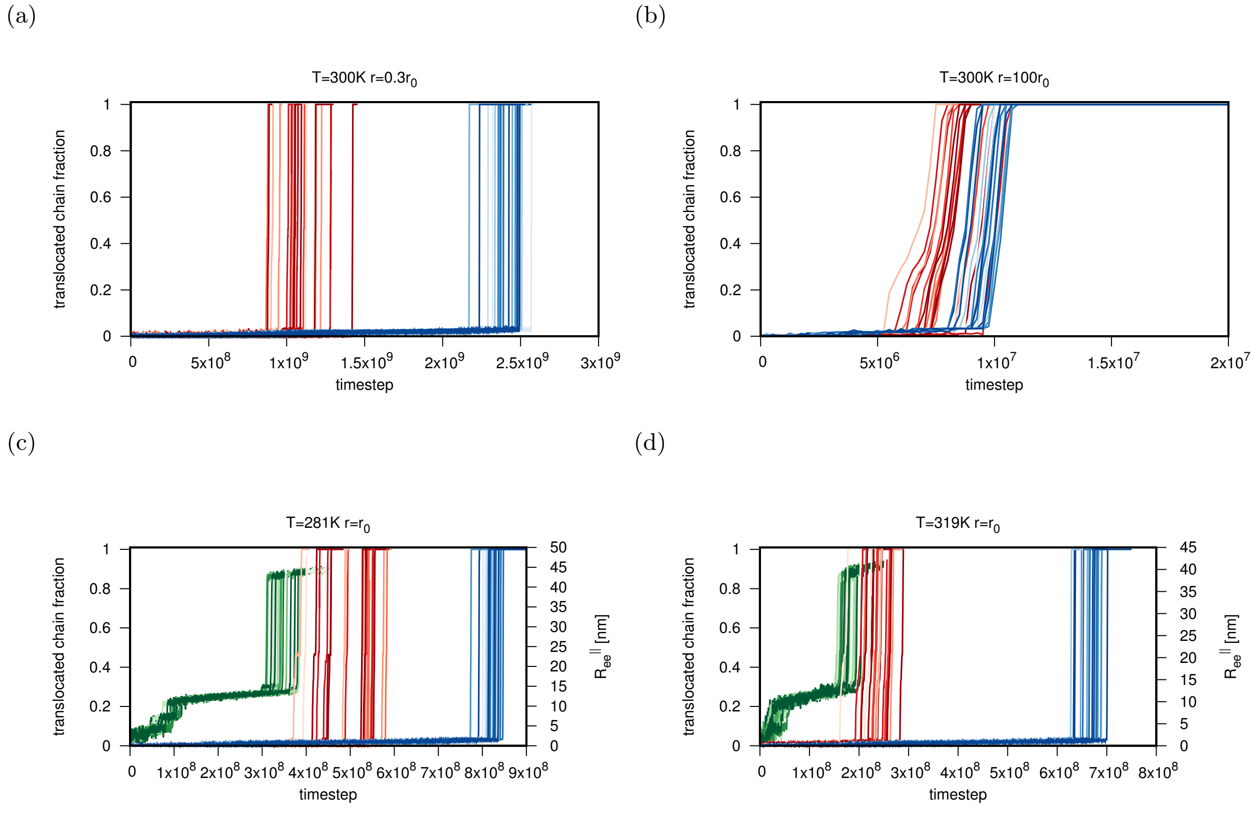

Supplementary Figure 4: Temporal trace of the translocated fraction of the Zika xrRNA for $5'$ (blue curves) and $3'$ (red curves) entries for different combinations of temperature, T, and ramping rates, r. Panels (c) and (d) additionally report the longitudinal end-to-end distance, $R_{ee}^{||}$ (green curves), as obtained in stretching simulations at the same values of T and r.

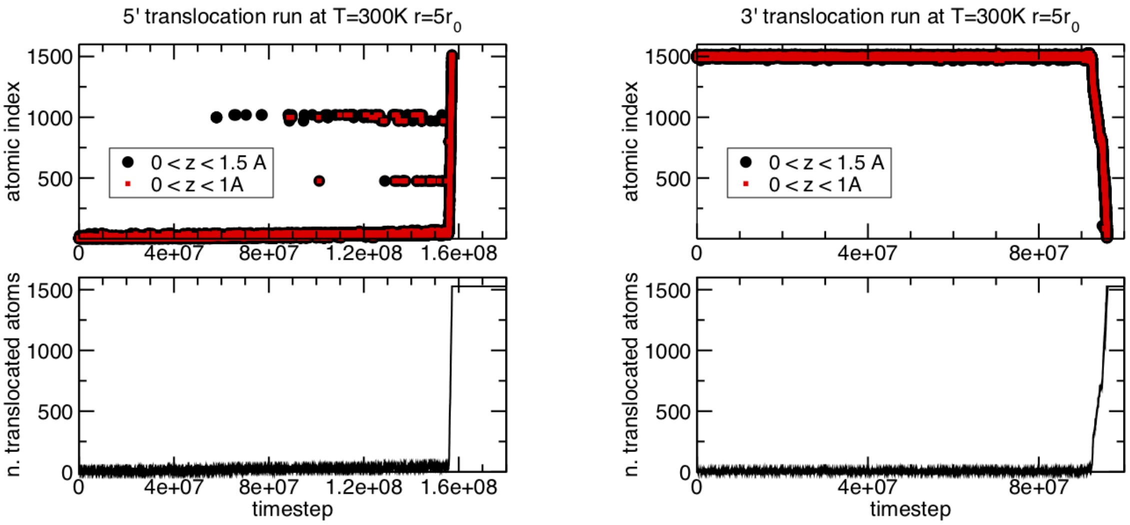

Supplementary Figure 5: (top) Temporal traces showing the indices of the atoms that during the translocation process have vertical coordinate $0 \leq z \leq 1.0$Åor $0 \leq z \leq 1.5$Å. Data are for a translocation run from either of the two ends at $T = 300$K and $r = 5\,r_0$. The two bands visible for $5'$ entries cover the ranges 969-1020 (corresponding to nucleotides A45-C48) and 474-477 (corresponds to nucleotide C22) which are eventually drawn in tight contact with the slab surface prior to the triggering of irreversible translocation. The number of translocated atoms is shown in the bottom panel for reference.

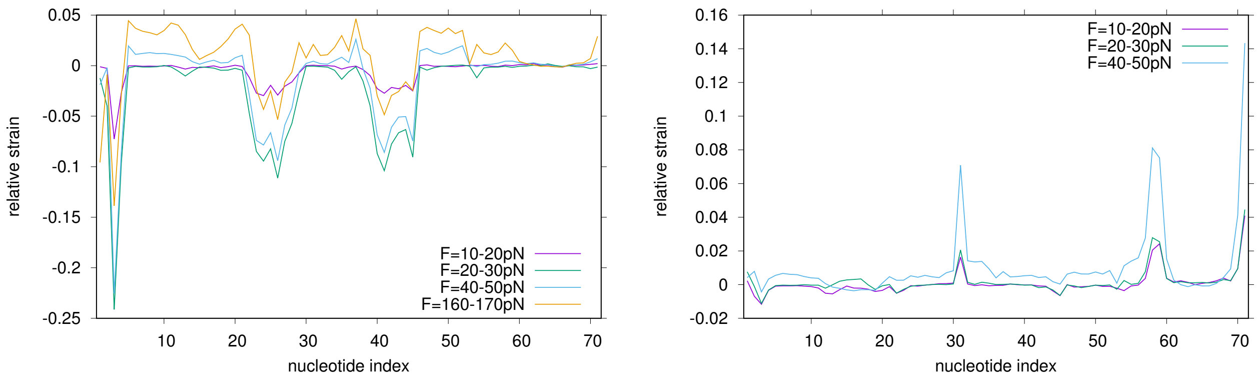

Supplementary Figure 6: Relative strain profile as a function of the nucleotide index, for translocations from the $5'$ (left panel) and $3'$ (right panel) ends, computed at the indicated force ranges in translocations at $T = 300$K and pulling rate equal to r_0.

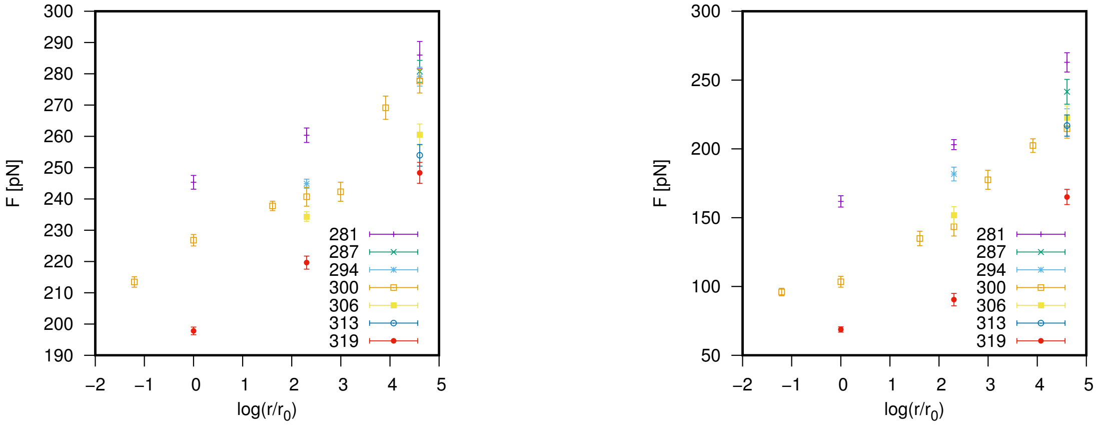

Supplementary Figure 7: Modal values of the critical (triggering) forces for 5′ (left) and 3′ (right) translocations for the 19 combinations of temperature (different colors) and force ramping rates, r/r_0.

F. Metadynamics simulations

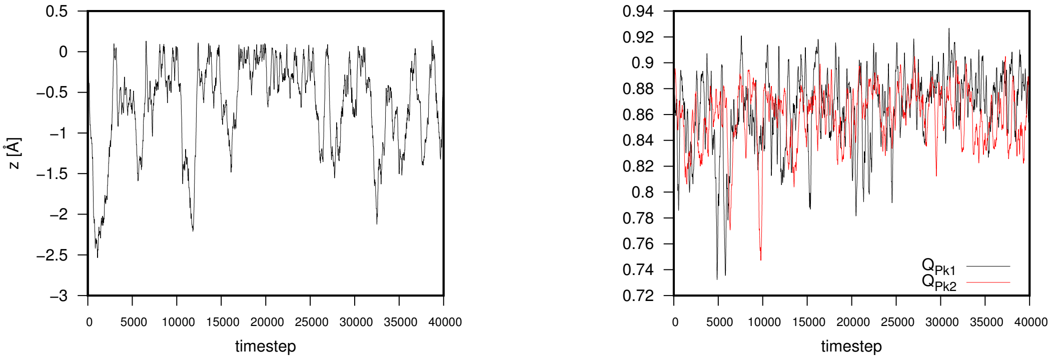

Supplementary Figure 8: Unbiassed trajectory of the three collective variables used for the metadynamics run. The root mean square fluctuations of the three variables is $RMSF_z = 0.5$Å, $RMSF_{Q_{Pk1}} = 0.052$ and $RMSF_{Q_{Pk2}} = 0.034$. The widths of the multivariate metadynamics Gaussians were set to values smaller than this threshold.

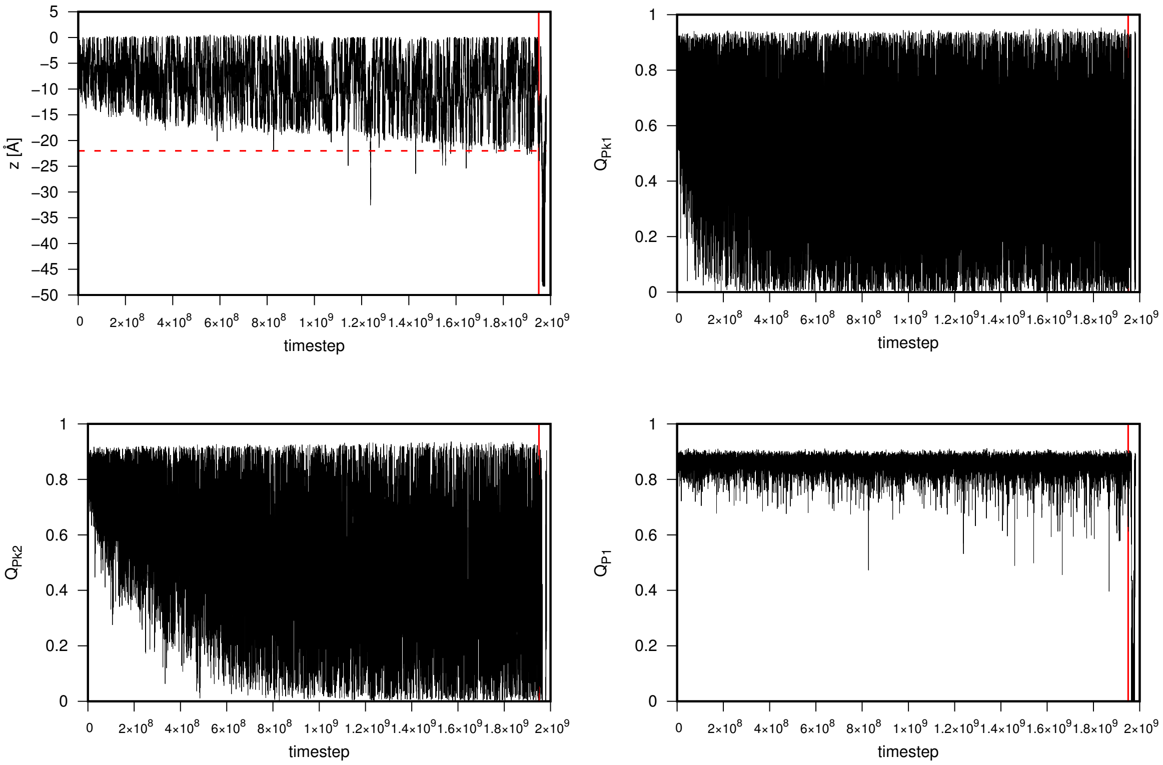

Supplementary Figure 9: Temporal evolution of the three collective variables z, Q_{Pk1} and Q_{Pk2} during a metadynamics run. The red (dashed) horizontal lines the pore insertion depth $z = -22$Å. At sufficiently long times, marked by the red (continuous) vertical line, helix P1 eventually breaks and, because its overlap Q_{P1} is not included in the collective variables, it is not rapidly reconstituted afterwards. Only the portion of trajectory prior to the breaking of P1 is used to reconstructed the free energy.

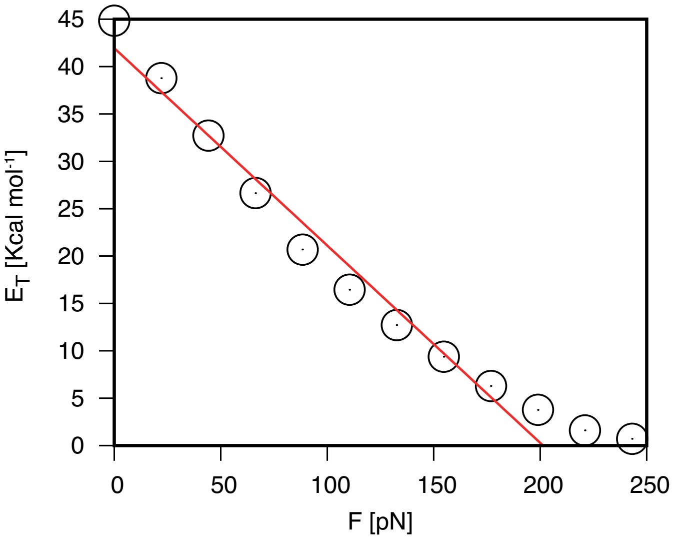

Supplementary Figure 10: An effective width of the free-energy barrier, can be defined in terms of the variation of the barrier height, E_T upon varying the force, F, $\Delta = -\partial E_T/\partial F$. The data points show the barrier height for the metadynamics free-energy profile at various values of the added force, F. The barrier height, E_T, was computed as the difference between the maximum and minimum values of the force-adjusted free energy for -19.5Å$\leq z \leq 0$. Linear interpolation of the central datapoints (red line) yields $\Delta = 13.4$Å, comparable to the reduced one of 19.5Å, corresponding to the difference of the z values to go from pore entrance to the triggering of translocation at the $5'$ end.

Supplementary References

[1] Colizzi, F. *et al.* Asymmetric base-pair opening drives helicase unwinding dynamics. *Proc. Natl. Acad. Sci. USA* **116**, 22471–22477 (2019).

[2] Yang, H. *et al.* Diffusion of trna inside the ribosome is position-dependent. *The Journal of chemical physics* **151**, 085102 (2019).
