# Supplementary material for: Directional translocation resistance of Zika xrRNA
Source: Nat Commun. 2020 Jul 27;11:3749. doi: 10.1038/s41467-020-17508-7 (PMC7385498; doi:10.1038/s41467-020-17508-7)
Supplement: Supplementary file 2 — Supplementary Software 1 [file 41467_2020_17508_MOESM2_ESM.zip › Source_Data_Simulation_setup_files/README.rtf]

23/03/2020“Directional translocation resistance of Zika xrRNA”by A. Suma, L. Coronel, G. Bussi and C. MichelettiDescription of setup files for MD and metadynamics simulationsThe directories:LAMMPS_setup_files_Stretching_T=300K_ramping-rate=r0_run1LAMMPS_setup_files_Translocation_3prime_end_T=300K_ramping-rate=r0_run1LAMMPS_setup_files_Translocation_5prime_end_T=300K_ramping-rate=r0_run1Metadynamics_on_5prime_endcontain the sample data files and input scripts for the ramped stretching and translocation LAMMPS simulations as well as for metadynamics runs based LAMMPS+COLVAR module.LAMMPS simulations require an executable compiled with custom extensions for the SMOG model and pore interactions. The source files for this extension are provided in the directory: Custom_LAMMPS_extension_for_pore_translocation.They need to be included in the "src" folder of the  22Aug18 version of LAMMPS to compile the appropriate lammps executable, lmp_mpi .MPI LAMMPS simulations are run with the typical command:mpirun -np $numberofprocessors location_to_folder/lammps-22Aug18/src/lmp_mpi -in input_script.lammpswhere input_script.lammps is the appropriate input file.The provided stretching and translocation examples are for the default ramping rate (r0) and temperature (T=300K in real units, T=95 in SMOG-LAMMPS units). The provided example for metadynamics is for a constant translocating force equal to 0.5 in SMOG-LAMMPS units.Atomistic simulations were carried out with the LAMMPS style “real”, where quantities are expressed with the following unitsdistance = Angstromsmass=grams/moletemperature=Kelvinenergy=Kcal/moleforce=Kcal/(mole Angstroms)time=femtosecondsFor the mapping between reduced simulation units and physical units see the Supporting information PDF. 
