# Supplementary material for: Directional translocation resistance of Zika xrRNA
Source: Nat Commun. 2020 Jul 27;11:3749. doi: 10.1038/s41467-020-17508-7 (PMC7385498; doi:10.1038/s41467-020-17508-7)
Supplement: Supplementary file 3 — Reporting Summary [file 41467_2020_17508_MOESM3_ESM.pdf]

nature research

Corresponding author(s): Manuscript: NCOMMS-20-10614A; Corr. auth.: Cristian Micheletti

Last updated by author(s): Jun 10, 2020

Reporting Summary

Nature Research wishes to improve the reproducibility of the work that we publish. This form provides structure for consistency and transparency in reporting. For further information on Nature Research policies, see our Editorial Policies and the Editorial Policy Checklist.

Statistics

For all statistical analyses, confirm that the following items are present in the figure legend, table legend, main text, or Methods section.

n/a	Confirmed	
☐	☒	The exact sample size (*n*) for each experimental group/condition, given as a discrete number and unit of measurement
☒	☐	A statement on whether measurements were taken from distinct samples or whether the same sample was measured repeatedly
☒	☐	The statistical test(s) used AND whether they are one- or two-sided<br>*Only common tests should be described solely by name; describe more complex techniques in the Methods section.*
☒	☐	A description of all covariates tested
☒	☐	A description of any assumptions or corrections, such as tests of normality and adjustment for multiple comparisons
☐	☒	A full description of the statistical parameters including central tendency (e.g. means) or other basic estimates (e.g. regression coefficient) AND variation (e.g. standard deviation) or associated estimates of uncertainty (e.g. confidence intervals)
☒	☐	For null hypothesis testing, the test statistic (e.g. *F*, *t*, *r*) with confidence intervals, effect sizes, degrees of freedom and *P* value noted<br>*Give P values as exact values whenever suitable.*
☒	☐	For Bayesian analysis, information on the choice of priors and Markov chain Monte Carlo settings
☒	☐	For hierarchical and complex designs, identification of the appropriate level for tests and full reporting of outcomes
☐	☒	Estimates of effect sizes (e.g. Cohen's *d*, Pearson's *r*), indicating how they were calculated

Our web collection on statistics for biologists contains articles on many of the points above.

Software and code

Policy information about availability of computer code

Data collection	The atomistic native-centric model was created with the SMOG webserver, using as input the PDB entry of Zika xrRNA. The traslocation and stretching simulations were carried out with the LAMMPS software package. Metadynamics simulations were carried out with LAMMPS and the COLVAR module. Setup simulation files are made available as a Supplementary Dataset. Conversion tools for converting input scripts from native SMOG GROMACS format to LAMMPS are made available on GitHub.
Data analysis	Translocation and stretching trajectories were analysed by monitoring the structural data reported in the figures (number of translocated nucleotides, end-to-end distance, overlap of various groups of native contacts). These structure-based quantities were computed with custom shell scripts or code. The metadynamics analysis was part of the COLVAR module output plus a standard thermodynamic integration based on custom code.

For manuscripts utilizing custom algorithms or software that are central to the research but not yet described in published literature, software must be made available to editors and reviewers. We strongly encourage code deposition in a community repository (e.g. GitHub). See the Nature Research guidelines for submitting code & software for further information.

Data

Policy information about availability of data

All manuscripts must include a data availability statement. This statement should provide the following information, where applicable:

- Accession codes, unique identifiers, or web links for publicly available datasets
- A list of figures that have associated raw data
- A description of any restrictions on data availability

The source data underlying Figures 1C, 2B, 3A, 3B, 4A and 5 are made available as Supplementary Dataset.

Field-specific reporting

Please select the one below that is the best fit for your research. If you are not sure, read the appropriate sections before making your selection.

- [x] Life sciences
- [ ] Behavioural & social sciences
- [ ] Ecological, evolutionary & environmental sciences

For a reference copy of the document with all sections, see nature.com/documents/nr-reporting-summary-flat.pdf

Life sciences study design

All studies must disclose on these points even when the disclosure is negative.

Sample size	Our study is theoretical/computational. The number of considered runs and conditions were set based on preconditioning runs were we assessed the varations of translocation times (from both ends) for different ramping protocols. Based on this, and the overall computational time available, we defined the number of different temperatures (T) and ramping rates (r) to consider, as well as the number of runs (20) for each (T,r) combination, which sufficed to identify the mean or modal translocation times with small uncertainty compared to the variations across T and r, as seen in the figures.
Data exclusions	Metadynamics data were used to reconstruct the free energy up to the breaking of helix P1, as discussed in Fig. S9.
Replication	n/a
Randomization	n/a
Blinding	n/a

Reporting for specific materials, systems and methods

We require information from authors about some types of materials, experimental systems and methods used in many studies. Here, indicate whether each material, system or method listed is relevant to your study. If you are not sure if a list item applies to your research, read the appropriate section before selecting a response.

Materials & experimental systems

n/a	Involved in the study	
☒	☐	Antibodies
☒	☐	Eukaryotic cell lines
☒	☐	Palaeontology and archaeology
☒	☐	Animals and other organisms
☒	☐	Human research participants
☒	☐	Clinical data
☒	☐	Dual use research of concern

Methods

n/a	Involved in the study	
☒	☐	ChIP-seq
☒	☐	Flow cytometry
☒	☐	MRI-based neuroimaging

April 2020
