# Supplementary material for: Directional translocation resistance of Zika xrRNA
Source: Nat Commun. 2020 Jul 27;11:3749. doi: 10.1038/s41467-020-17508-7 (PMC7385498; doi:10.1038/s41467-020-17508-7)
Supplement: Supplementary file 4 — Source Data [file 41467_2020_17508_MOESM4_ESM.zip › Source_Data_Figures/README.rtf]

07/05/2020“Directional translocation resistance of Zika xrRNA”by A. Suma, L. Coronel, G. Bussi and C. MichelettiDescription of data filesThe folders named:Fig1CFig2BFig3AFig3BFig4AFig5contain the data used in the namesake figures of the manuscript, as detailed below.________________Fig1CThe folder contains the data file Fig1c.dat containing the native contact map of the Zika xrRNA. The data file is organised in three columns corresponding to:1) nucleotide index, i2) nucleotide index, j3) number of native contacts between atoms of nucleotides i and j.________________Fig2B The folder contains the three following subfolders:3'translocation-rate_r0-T300K  and  5’translocation-rate_r0-T300K ,Each containing 20 data files r_*_translocated_portion.dat related to the red and blue curves in Fig.2B, respectively. Each data file is organised in two columns corresponding to:	1) simulation timestep; convert to microseconds by multiplying by 1.13e-9		2) number of translocated atoms; divide by 1527 to obtain the translocated fractionstretching-rate_r0-T300K ,containing 20 data files r_*_Ree_parallel.dat related to the green curves in Fig.2B. Each data file is organised in two columns corresponding to:	1) simulation timestep; convert to microseconds by multiplying by 1.13e-9			2) longitudinal component of the end-to-end distance, in Angstroms ________________Fig3AThe folder contains the two data files, 3’end-translocation-data.dat  and 5’end-translocation-data.dat related to Fig.3A of the paper. Each data file is organised in four columns corresponding to:	1) force ramping rate, in simulation units. In these units r0=1.37e-9	2) temperature, in simulation units; convert to physical units (K) by multiplying by alpha=3.16				3) most probable translocation force, in simulation units; convert to physical units (pN) by multiplying by alpha*69.5pN =210pN			4) error estimate on the most probable translocation force________________Fig3B The folder contains the data files WT-5’end.dat DelPk1.dat DelPk2.dat DelPk1-2.dat WT-3'end.dat related to Fig.3B. The data file names indicate their corresponding mutation, and are organised in 2 columns corresponding to:	1) run id	2) translocation force, in simulation units; convert to physical units (pN) by multiplying by alpha*69.5pN =210pN________________Fig4A The folder contains two data files, 3’translocation-kymograph.dat and 5'translocation-kymograph.dat related to Fig.4A. Each data file is organised in 3 columns corresponding to:	1) interval of force ramping stage (1, 2, 3 and 4 stand, respectively for, 10-20pN,  20-30pN, 40-50pN and 160-170pN)	2) index of nucleotide, i	3) relative strain (%) of the corresponding native interaction network.	________________Fig5 The folder contains the data file metadaynamics-free-energy.pmf related to the free energy profile of Fig. 5. The data file is organised in 4 columns corresponding to:	1) pore insertion depth z, in Angstrom	2) fraction of native contacts Q_Pk1	3) fraction of native contacts Q_Pk2	4) corresponding free energy in simulation units; convert to physical units (Kcal/mol) by multiplying by alpha=3.16.	________________
